# Prevalence of Radial Artery Variants and Their Relationship with Clinical Considerations of the Antebrachial Region: Systematic Review and Meta-Analysis

**DOI:** 10.3390/diagnostics15232984

**Published:** 2025-11-24

**Authors:** Juan Sanchis-Gimeno, Jessica Paola Loaiza-Giraldo, Yael Alruiz, Maximiliano Vergara, Maria Fernanda Navia, Camila Roman, Alejandra Suazo-Santibañez, Pablo Nova-Baeza, Mathias Orellana-Donoso, Gustavo Oyanedel-Amaro, Macarena Rodriguez-Luengo, Alejandro Bruna-Mejias, Juan José Valenzuela-Fuenzalida, Jose E. León-Rojas, Guinevere Granite

**Affiliations:** 1 GIAVAL Research Group, Department of Anatomy and Human Embryology, Faculty of Medicine, University of Valencia, 46001 Valencia, Spain; juan.sanchis@uv.es; 2 Facultad de Ciencias de la Salud, Unidad Central del Valle del Cauca (UCEVA), Tuluá 763022, Valle del Cauca, Colombia; 3 Departamento de Morfología, Facultad de Medicina, Universidad Andrés Bello, Santiago 8370146, Chile; 4 Faculty of Health and Social Sciences, Universidad de Las Américas, Santiago 8370040, Chile; 5 Escuela de Medicina, Universidad Finis Terrae, Santiago 7501015, Chile; 6 Facultad de Medicina y Ciencia, Universidad San Sebastián, Lota 2465, Santiago 7510157, Chile; 7 Facultad de Ciencias de la Salud, Universidad Autónoma de Chile, Santiago 8910060, Chile; 8 Departamento de Ciencias y Geografía, Facultad de Ciencias Naturales y Exactas, Universidad de Playa Ancha, Valparaíso 2360072, Chile; 9 Departamento de Ciencias Químicas y Biológicas, Facultad de Ciencias de la Salud, Universidad Bernardo O’Higgins, Santiago 8370993, Chile; 10 Cerebro, Emoción y Conducta (CEC) Research Group, Escuela de Medicina, Universidad de las Américas (UDLA), Quito 170503, Ecuador; 11 Department of Surgery, Uniformed Services University of the Health Sciences, Bethesda, MD 20814, USA; guinevere.granite@usuhs.edu

**Keywords:** anatomy, radial artery, radial artery variation, clinical anatomy, anatomical variation

## Abstract

**Background:** The radial artery (RA) is one of the terminal branches of the brachial artery, extending along the lateral forearm, crossing the anatomical snuffbox, and contributing to the palmar arches. Anatomical variations in the RA are of great clinical relevance due to their implications in procedures such as transradial catheterization, arterial cannulation, and bypass grafting. These variants may alter the course, branching pattern, or origin of the vessel, potentially increasing procedural complexity and the risk of iatrogenic injury. In critically ill patients and in surgical or interventional settings, accurate identification of RA anatomy is essential. The objective of this study was to systematically identify and describe RA variants reported in the scientific literature and to analyze their clinical relevance. **Methods:** A systematic search was conducted across six electronic databases: Medline, Scopus, Web of Science, Google Scholar, Cumulative Index to Nursing and Allied Health Literature (CINAHL), and Latin American and Caribbean Literature in Health Sciences (LILACS), covering publications up to July 2025. Eligible studies included anatomical, radiological, and surgical investigations reporting RA variants. Study quality was evaluated using the Assessment of Quality in Anatomical Studies (AQUA) tool. Quantitative synthesis was performed using a random-effects model to estimate the pooled prevalence of RA variants and subgroup differences. Twenty-three studies met the inclusion criteria, and eleven were included in the meta-analysis, encompassing a total of 6320 participants. **Results:** Radial artery variants were categorized into three main types: variations in origin, course, and branching pattern. The pooled global prevalence of RA variants was 12% (95% CI: 6–18%), with substantial heterogeneity (I^2^ = 97.7%). Higher prevalence was found in imaging-based studies (14%) compared with donor-based studies (12%). Sex-based subgroup analysis revealed a higher prevalence in females (18%; CI: 9–28%) compared with males (3%; CI: 3–4%), with moderate heterogeneity (I^2^ = 61.3%). Regionally, European populations demonstrated a higher prevalence (20%) than Asian populations (11%), both showing high heterogeneity (I^2^ > 98%). Notably, only one study from the Americas and none from Africa or Oceania were identified, representing a major geographical limitation in the available data. The findings of this study highlight the considerable variability in RA anatomy across populations. Such variations hold significant clinical importance, particularly in the context of transradial interventions, arterial cannulation, and reconstructive procedures where vascular integrity is critical. The high degree of heterogeneity observed may reflect differences in population genetics, sample size, and imaging or dissection methodologies. The limited representation of certain regions underscores the need for further anatomical and radiological studies to obtain a more comprehensive understanding of global RA variability. Preoperative or pre-procedural imaging using Doppler ultrasonography or computed tomography angiography is recommended to identify anomalous patterns and minimize iatrogenic complications. **Conclusions:** Radial artery variants are frequent and diverse. Their recognition is fundamental for the safety and success of invasive and surgical procedures in the upper limb. A standardized approach to vascular evaluation, particularly through preoperative imaging, is essential to improve procedural outcomes and reduce the risk of arterial injury in clinical practice.

## 1. Introduction

The radial artery (RA) represents one of the two principal terminal branches of the brachial artery (BA), the other being the ulnar artery (UA). The BA bifurcates within the cubital fossa, approximately at the level of the radial neck, giving rise to both arteries. Following its origin, the RA courses distally along the lateral aspect of the forearm, situated between the brachioradialis and flexor carpi radialis muscles. Distal to the wrist joint, the RA and UA form anastomotic networks contributing to the superficial and deep palmar arches, ensuring adequate perfusion of the hand [1,2,3,4].

The embryological development of the RA remains a subject of debate. Current hypotheses propose that the RA originates through progressive sprouting from a primitive capillary plexus, undergoing angiogenesis and vascular remodeling regulated by intricate interactions among molecular activators and inhibitors within signaling pathways. By approximately the 44th day of gestation, most arterial structures of the forearm are established, although the distal segment of the RA develops later. This delayed maturation may account for the frequent anatomical variations observed in this vessel, although the exact mechanisms remain unclear. The RA gives off multiple branches, including the radial recurrent artery, muscular branches along the radial border, and the palmar carpal and superficial palmarbranches in the forearm; the dorsal carpal branch and first dorsal metacarpal artery at the wrist; and, in the hand, the princeps pollicis artery, radialis indicis artery, and the deep palmar arch. The RA receives sympathetic innervation predominantly from spinal segments C6 and C7, supplying the posterolateral compartment of the forearm, the elbow joint, the carpus, and the lateral digits [3,5,6].

A wide spectrum of anatomical variations has been documented in the arterial system of the upper limb, both of physiological and pathological relevance. Specifically, Ostojić et al. (2015) reported a prevalence of 8.8% of RA variations among patients undergoing transradial cardiac catheterization, including anomalies in the origin, morphology, course, branching pattern, tortuosity, and termination of the artery [2,3]. The clinical significance of such variations is substantial, as they may influence outcomes in diverse medical and surgical procedures, including arterial blood gas sampling, radial artery cannulation, arteriovenous fistula creation, and coronary interventions, as well as emergency ligation of the RA. Moreover, understanding RA anatomy is essential for accurate vital sign assessment, given that the radial pulse palpable proximal to the wrist crease, lateral to the flexor carpi radialistendon, or within the anatomical snuffbox is routinely used for hemodynamic evaluation. The Allen test serves as a simple bedside method to assess collateral perfusion of the hand by sequentially compressing both the RA and UA, inducing temporary pallor, and then releasing one artery at a time to verify the restoration of blood flow [1].

The objective of this study was to systematically identify and describe the radial artery variants reported in the scientific literature and to analyze their clinical and surgical relevance.

## 2. Methods

### 2.1. Protocol and Registration

A systematic review and meta-analysis were carried out to determine the frequency of variations in the origin, course, and branching patterns of the radial artery (RA), as well as to analyze their relationship with reported clinical manifestations. The protocol followed the recommendations of the Preferred Reporting Items for Systematic Reviews and Meta-Analyses (PRISMA) statement [7]. The study was prospectively registered in the International Prospective Register of Systematic Reviews (PROSPERO) under the registration number CRD42024520734.

### 2.2. Eligibility Criteria

The studies included in this review met specific eligibility criteria. The population comprised cadaveric dissections and in vivo imaging studies that documented variations in the radial artery (RA). The outcomes focused on determining the prevalence of these variants and exploring their potential relationship with forearm pathologies. Anatomical variations were classified and described according to classical anatomical references and previously established classification systems. Only original research articles and case reports involving human samples, published in English in peer-reviewed journals indexed in the selected databases, were included, whereas letters to the editor and other non-peer-reviewed publications were excluded.

### 2.3. Electronic Search

A comprehensive literature search was conducted across the following databases: MEDLINE (via PubMed), Web of Science, Google Scholar, the Cumulative Index to Nursing and Allied Health Literature (CINAHL), Scopus, and Latin American and Caribbean Health Sciences Literature (LILACS), covering all records from their inception to April 2025. The search strategy incorporated combinations of the following terms: “Anatomy radial artery,” “variations radial artery,” “aberrant radial artery,” “clinical anatomy,” “blood supply forearm,” and “anatomical variations,” applying the Boolean operators AND, OR, and NOT. The detailed search strategies for each database are presented in Table 1. Additional or extended search details are provided in the Supplementary Materials. 

### 2.4. Study Selection

**Figure 1:**
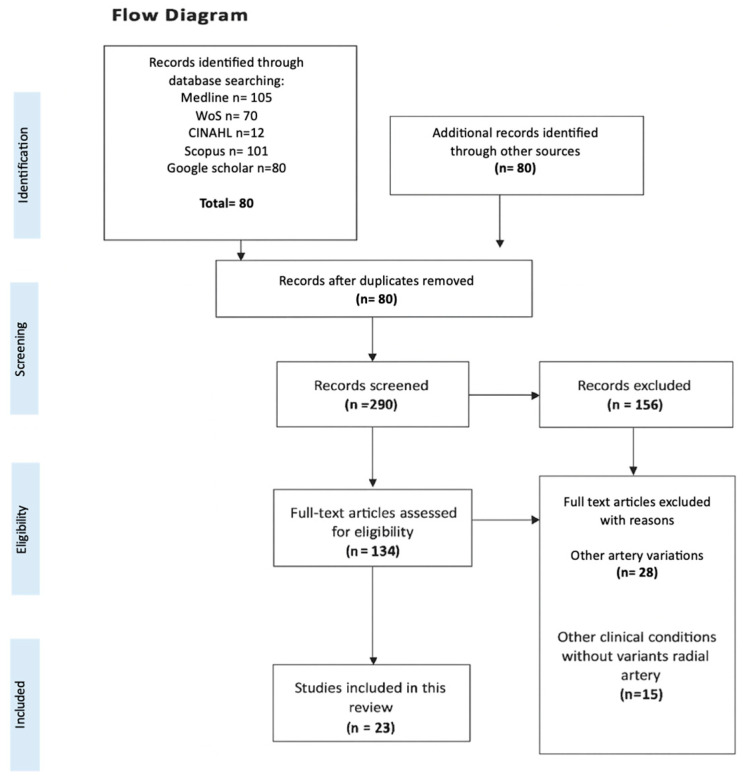
Flow diagram search. Explanation of the selection process and data collection.

The titles and abstracts of all references identified through the database searches were screened for relevance, and duplicate records were removed. Studies describing variations in arteries other than the radial artery, as well as those addressing clinical conditions unrelated to RA variants, were excluded. The inter-evaluator reliability was assessed using the Kappa statistic, which yielded a value of 0.66 (Figure 1).

### 2.5. Data Collection Process

The following data were extracted from the original studies: (i) author and year of publication, (ii) geographic region, (iii) age and sex of the subjects, (iv) prevalence, (v) clinical history, (vi) symptoms, (vii) clinical circumstances, (viii) types of radial artery (RA) variants, (ix) characteristics of the arterial variations, (x) other potentially relevant features, and (xi) clinical implications.

### 2.6. Assessment of the Methodological Quality of the Included Studies

Quality assessment was performed using the methodological quality assurance tool for anatomical studies (AQUA) proposed by the International Evidence-Based Anatomy Working Group (IEBA) [8].

### 2.7. Statistical Methods

The data extracted for the meta-analysis were analyzed using R statistical software (version 4.4.0) (accessed in July 2025) to estimate the prevalence of morphological variants of the radial artery (RA). Summary data were combined using the DerSimonian–Laird random-effects model with a Freeman Tukey double arcsine transformation. A random-effects approach was selected due to the high heterogeneity observed in the prevalence estimates of RA variants.

Heterogeneity among the included studies was evaluated using Pearson’s chi-squared test and the I^2^ statistic. For the chi-squared test, a *p*-value of 0.10 was considered statistically significant, as recommended by the Cochrane Collaboration. The I^2^ values, interpreted with a 95% confidence interval (CI), were classified as follows: 0–40% indicating low or no heterogeneity, 30–60% moderate heterogeneity, 50–90% substantial heterogeneity, and 75–100% considerable heterogeneity [9].

To assess the presence of a small-study effect where smaller studies may report different effects compared to larger ones a DOI plot with the Luis Furuya-Kanamori (LFK) index was generated [10,11].

### 2.8. Subgroup Analysis

The sample was divided into multiple subgroups to evaluate potential differences in radial artery (RA) variants across the population. Subgroup analyses were performed based on study or sample type, geographic region (continent), sex, side, and laterality. For study/sample type, the data were categorized into imaging studies and donor specimens. Geographic regions represented included Asia, Europe, and the Americas. Laterality analyses compared the frequency of bilateral versus unilateral RA variants, and left- versus right-sided variants were also assessed. Separate statistical analyses were conducted for each subgroup.

## 3. Results

### 3.1. Included Articles

A total of 23 studies [2,12,13,14,15,16,17,18,19,20,21,22,23,24,25,26,27,28,29,30,31,32,33] were included in the analysis (Figure 1 and Table 2), encompassing approximately 7479 individuals. Both male and female participants were reported in most studies, with one study not specifying sex [25]. Of the total sample, approximately 5421 were male and 1936 were female, with a mean age of 61.2 years. Regarding geographic distribution, 12 studies were conducted in Asia [2,12,14,21,22,23,27,28,29,30,32], six in Europe [15,19,20,24,29,30], and four in the Americas [13,17,25,33], while no studies from Africa or Oceania were included.

### 3.2. Variant Descriptions and Anatomy

#### 3.2.1. Variants in the Origin of the Radial Artery

A wide range of anatomical variations in the origin of the radial artery (RA) was reported in the included studies. The RA most commonly originated from the medial aspect of the upper third or the upper lateral portion of the brachial artery (BA). Other reported variations included a bifurcated BA proximal to the intercondylar line of the humerus, and in one case, a radioulnar loop in which the RA arose from the ulnar artery (UA) instead of the BA [2,12,13,23].

**Figure 2:**
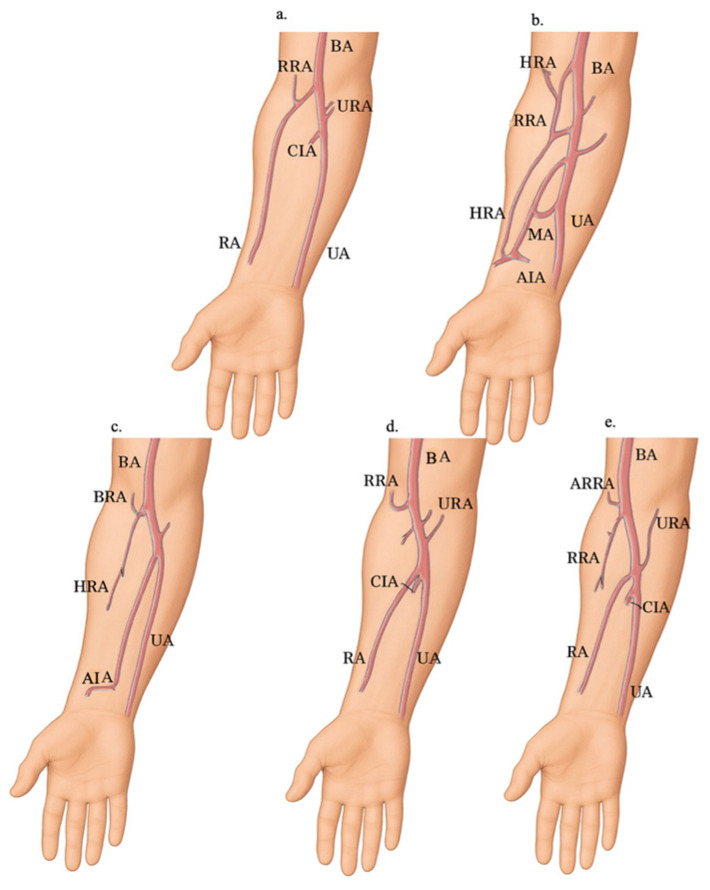
Selected anatomical variations in the radial artery (RA) in the forearm. (**a**) Typical course of the RA. (**b**) Rudimentary RA with three anastomotic roots: the superior root originating from the axillary artery, the middle root from the median artery, and the inferior root from the anterior interosseous artery. In this case, the RA was present along its entire course. Adapted from Gruber (1870a) [34]. (**c**) RA replaced by an atypical branch of the anterior interosseous artery, maintaining the original Gruber (1870b) [34] classification in the labeling. (**d**) Low division and trifurcation of the brachial artery, as described by Vollala et al. (2008) [32]. (**e**) Low-origin RA coexisting with a double recurrent radial artery, as reported in this study. Abbreviations: AIA: Anterior interosseous artery; ARRA: Accessory radial recurrent artery; BA: Brachial artery; CIA: Common interosseous artery; HRA: Hypoplastic (rudimentary) radial artery; MA: Persistent median artery; RA: Radial artery; RRA: Radial recurrent artery; UA: Ulnar artery; URA: Anterior and posterior ulnar recurrent arteries (in panel (**e**), a single ulnar recurrent artery is illustrated, as described in this report).

Less frequently observed variants included: a low-origin RA passing deep to the pronator teres muscle with a double recurrent RA; a rudimentary RA with three anastomotic roots arising from the axillary artery (AA); RA replaced by an atypical branch of the anterior interosseous artery; a low division of the BA resulting in trifurcation; anastomoses between the brachioradial artery and the median or anterior interosseous arteries; anastomoses between the RA and the superficial median artery; a thin, deep RA penetrating the tendons of the anatomical snuff-box; and a superficial palmar branch of the superficial RA supplying the thenar muscles [13,23,24,30,32] (Figure 2).

#### 3.2.2. Variants in the Course of the Radial Artery

Regarding the course of the RA, it is located between the tendon of the brachioradialis muscle and the flexor carpi radialis muscle, following the anatomical snuff-box. Some atypical topographic relationships are presented below such as superficial RA that runs over the tendons and thin deep RA and penetrates the tendons of the anatomic snuff-box, superficial palmar branch of the superficial RA to thenar muscles, a trifurcation of the BA in which the RA follows an abnormal course passing deep to the pronator teres muscle, and an aberrant RA in a left arm of a patient with Klippel-Feil syndrome who had progressive atrophy of the left thenar eminence (Figure 2) [14,16,31,32].

### 3.3. Analysis of Prevalence and Subgroups

**Figure 3:**
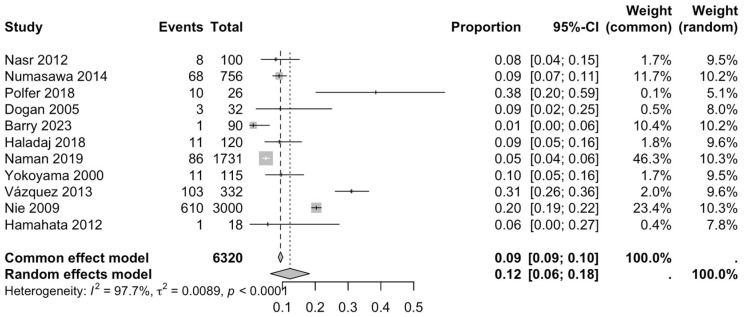
Forest plot of the total sample of RA prevalence variants [2,12,13,15,19,20,22,23,24,29,30].

Eight proportion forest plots were created to calculate the prevalence of RA variants in the studies included in the present systematic review. Eleven studies [2,12,13,15,19,20,22,23,24,29,30] were included for the calculation of the prevalence of the RA variants, presenting a global prevalence of 12% (CI: 6–18%) (Figure 3). The heterogeneity of the samples included was 97.7%.

**Figure 4:**
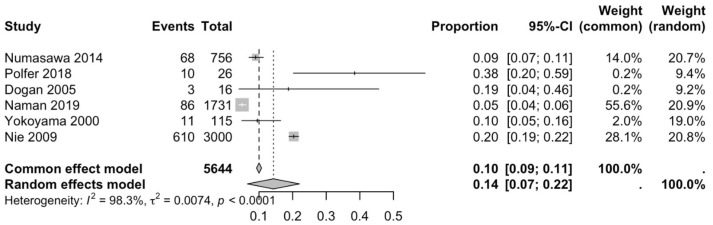
Forest plot of the prevalence of RA variants in imaging/surgical studies [12,13,15,22,23,29].

**Figure 5:**
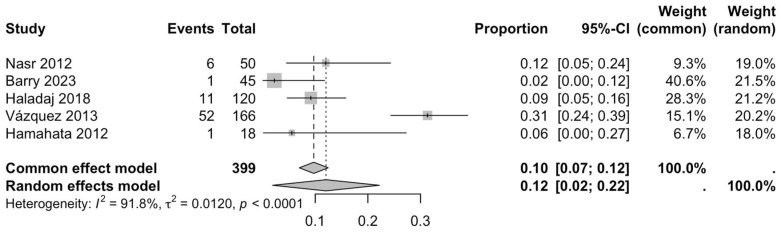
Forest plot of the prevalence of RA variants in donor studies [2,19,20,24,30].

**Figure 6:**
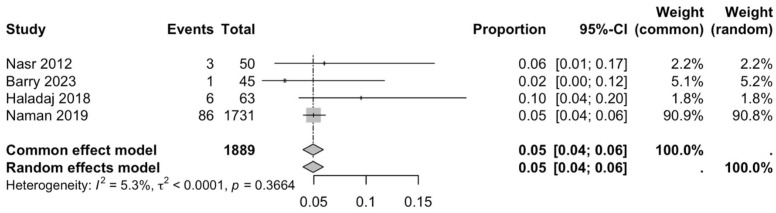
Forest plot of RA variant prevalence in right-sided samples [2,19,20,22].

**Figure 7:**
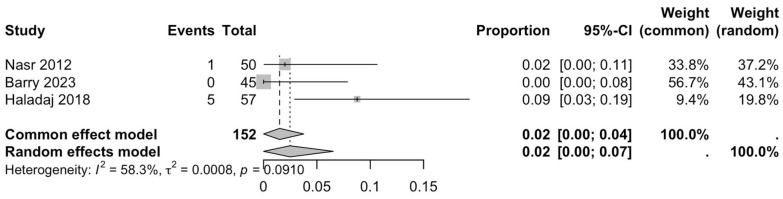
Forest plot of RA variant prevalence in left-sided samples [2,19,20].

**Figure 8:**
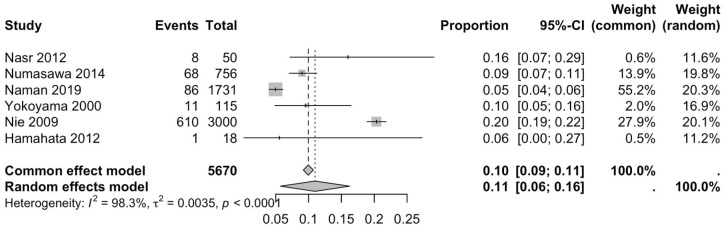
Forest plot of RA variant prevalence in Asian samples [2,12,22,23,29,30].

**Figure 9:**
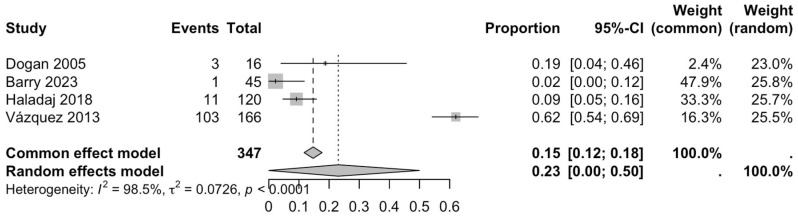
Forest plot of RA variant prevalence in European samples [15,19,20,24].

**Figure 10:**
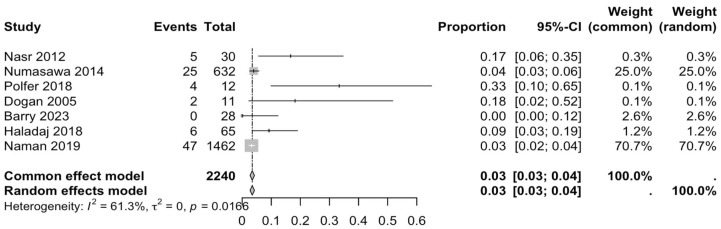
Forest plot of RA variant prevalence in male subjects [2,12,13,15,19,20,22].

**Figure 11:**
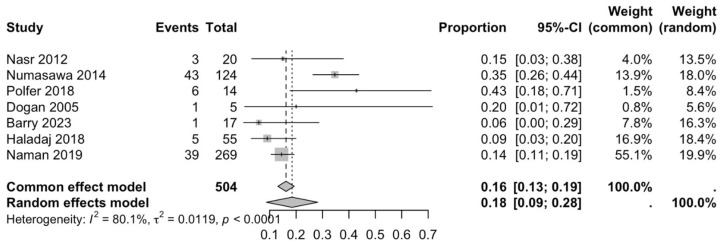
Forest plot of RA variant prevalence in female subjects [2,12,13,15,19,20,22].

The first subgroup included six studies with living subjects evaluated through imaging or surgical reports [12,13,15,22,23,29] were included in this analysis, with a prevalence of 14% (CI: 7% to 22%) and a heterogeneity of 98.3% (Figure 4). The second subgroup included donor evaluation in five studies [2,19,20,24,30] presenting a prevalence of 12% (CI: 2% to 22%) and a heterogeneity of 91.8% (Figure 5). The third subgroup analyzed was the laterality of the RA variants. The right-sided distribution of the median nerve was reported in four studies [2,19,20,22], with a prevalence of 5% (CI: 4–6%) and the heterogeneity among studies was 5.3% (Figure 6). For the left-sided distribution of the RA, three studies were included [2,19,20], showing a prevalence of 2% (CI: 0% to 7%) and the heterogeneity was 53.8% (Figure 7). Only one study was included for the bilateral distribution of the RA variations [2]. The fourth subgroup analysis was for the continents from which the included studies were conducted. Six studies from Asia were included [2,12,22,23,29,30], presenting a prevalence of 11% (CI: 6–16%) and a heterogeneity of 98.3% (Figure 8). From Europe, four studies were included [15,19,20,24], presenting a prevalence of 23% (CI: 0–50%) and a heterogeneity of 98.5% (Figure 9). From America, only one study was included [13]. Prevalence studies from Oceania and Africa were not included. Finally, the subgroup analysis performed for the sex of the included subjects, seven studies showed male individuals with the variant [2,12,13,15,19,20,22] which presented a prevalence of 3% (CI: 3–4%) and a heterogeneity of 61.3% (Figure 10). Conversely, seven studies reported females with RA variants [2,12,13,15,19,20,22], presenting a prevalence of 18% (CI: 9% to 28%) and heterogeneity of 80.1% (Figure 11).

### 3.4. Risk of Bias of Included Articles

**Figure 12:**
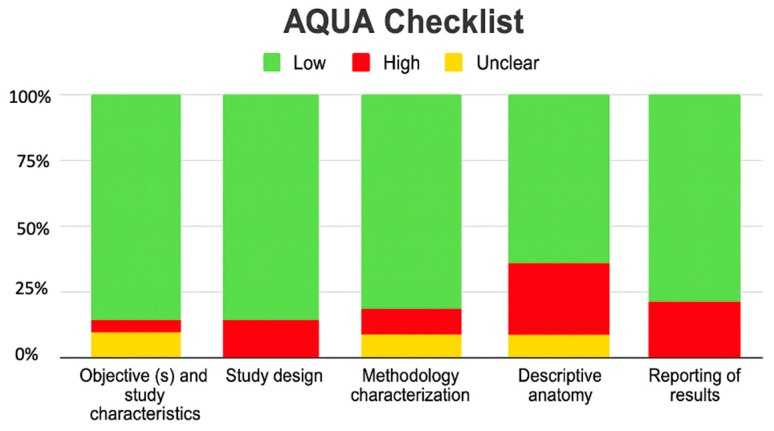
Risk of bias with AQUA checklist.

All studies met the assessment criteria using the AQUA checklist for anatomical studies, which assessed bias in five domains. Among the included studies, six studies were at high risk of bias [2,14,17,19,21,22] mainly in the domains of outcome reporting and descriptive anatomy, where the highest amount of bias occurred (Figure 12).

## 4. Discussion

This review aimed to identify the principal variations in the radial artery (RA), including those at its origin, along its course, and at its termination in the formation of the palmar arch. The results indicated that variations in the origin of the RA were more frequently observed than variations along its course or at its termination.

These findings have important clinical implications, as they can inform procedures such as catheterization, vascular imaging, and the administration of certain medications, helping to ensure a safe approach and minimize potential complications, including bleeding. Therefore, a thorough understanding of RA variants is essential for clinicians performing these interventions.

### 4.1. Previous Studies

The study by Ostojić et al. (2015) reported 8.8% of anatomical variations in patients undergoing transradial cardiac catheterization, which the most frequent was high origin of the RA (5.1%) under the pronator teres muscle, including congenital absence of the RA [3]. Also, Marchese et al. (2025) reported variations in the origin of RA in 9.2% of a total of 120 donors, of these the origin was in the axillary cavity in 18.1%, and 81.8% in the medial bicipital groove [1].

The study by Hoffman et al. (2020) [35] aimed to conduct systematic review of the literature on anatomical abnormalities of the RA that could affect harvesting a radial forearm flap for reconstructive surgery. The main findings included variations in the RA, such as accessory branches (0.5%), stenosis (1.3%), hypoplasia (1.9%), tortuosity (4.3%), and variations in origin (5.6%). No complications were reported; however, during flap harvesting. This review included 50 studies [35].

### 4.2. Anatomical Characteristics

As previously mentioned, the RA usually originates in the lower part of the cubital fossa after the bifurcation of the BA. One of the most frequent is the high bifurcation of the BA or a BRA, originating in the axillary cavity or the medial bicipital groove [1]. Other origins have been described in the upper medial or lateral third of the BA, proximal to the intercondylar line of the humerus [12,22]. Cases where the RA originates from the UA, that form a radioulnar loop, have also been documented [12,22,23,29]. Other less common variants include trifurcation of the BA, rudimentary RA, anastomosis with median or interosseous arteries, and even congenital absence of the RA [20,32]. Regarding its course, the RA generally runs between the brachioradialis muscle and flexor carpi radialis muscle, but it can also present with superficial, deep, or aberrant courses, as in Klippel-Feil syndrome [31] or variations in the anatomical snuff-box [14,16].

### 4.3. Forest Analysis

The global prevalence of the radial artery (RA) variant identified in this analysis was 12% (CI: 6–18%), calculated using the Freeman Tukey transformation method [36]. Although the heterogeneity was notably high (I^2^ = 97.7%) (Figure 3), this finding remains valuable as it derives from a broad compilation of studies encompassing diverse populations and methodologies, thereby providing a more realistic reflection of anatomical variability across clinical and demographic contexts. When comparing the prevalence of RA variants observed in imaging or surgical studies with those reported in anatomical donors, the values were nearly equivalent. This similarity may be attributed to the typically asymptomatic nature of these anatomical variations. Regarding laterality, the data revealed no significant differences between the right and left sides, suggesting the absence of a clear predominance in anatomical distribution. It is worth highlighting that one of the included studies analyzed only right upper limbs for a specific procedure, while another reported a bilateral variant, which precludes comparative analysis [2]. Regional comparisons demonstrated marked differences in prevalence. In Europe, the estimated prevalence was 23%, accompanied by heterogeneity comparable to the overall analysis (I^2^ = 98.5%) (Figure 9). In contrast, studies conducted in Asia reported a prevalence of 11% (CI: 6–16%) with similarly high heterogeneity (I^2^ = 98.3%) (Figure 8). These substantial regional discrepancies may be explained by demographic differences among the study populations and the distinct methodologies employed for anatomical assessment. The scarcity of studies from the Americas only one identified and the absence of data from Africa and Oceania represent important limitations, underscoring the need for future research to address these geographic gaps in the literature. Sex-based analysis revealed a higher prevalence in females (18%, CI: 9–28%) (Figure 11) compared with males (3%, CI: 3–4%) (Figure 10). The lower male prevalence, together with moderate heterogeneity (I^2^ = 61.3%), may suggest a more stable arterial configuration and reduced susceptibility to anatomical variation among men. However, this disparity could also be influenced by factors such as the smaller female sample sizes in certain studies [22], potential sampling bias, or methodological inconsistencies across investigations.

### 4.4. Clinical Considerations

The radial artery (RA) plays a pivotal role in a wide range of medical and surgical procedures; however, anatomical variations in its origin, course, or diameter can pose significant clinical challenges, affecting the safety and outcomes of interventions such as catheterization, coronary artery bypass grafting (CABG), and reconstructive surgeries [2,30]. These variations may contribute to complications including asymptomatic occlusion, arterial spasm, pseudoaneurysm formation, vessel perforation, and hematoma. More severe outcomes can include compartment syndrome, brachial artery (BA) perforation, and catheter entrapment. Accurate identification of such variations often relies on imaging techniques, including Doppler ultrasound and multidetector computed tomography (MDCT), which are crucial not only for detecting anatomical variants but also for identifying coexisting vascular pathologies such as calcifications or stenoses [18,23].

A hypoplastic RA can alter blood flow distribution and increase the risk of aneurysm formation due to chronically elevated hemodynamic stress [18]. Additionally, variations such as RA tortuosity are frequently associated with cardiovascular risk factors, including advanced age, elevated body mass index, hypertension, dyslipidemia, and smoking [21]. High-origin RA variants may compromise digital perfusion, increasing the risk of ischemic complications and thrombosis, which can be exacerbated by comorbidities such as scleroderma [13]. The presence of a brachioradial artery has been shown to contribute significantly to tortuosity, thereby increasing the likelihood of procedural failure during transradial catheterizations [20]. In these cases, alternative vascular access routes, such as transulnar, contralateral radial, brachial, or femoral approaches, have been employed effectively and safely [12,22].

For cannulation procedures, the RA remains a preferred site for blood pressure monitoring, arterial blood gas analysis, and various applications in anesthesia, emergency medicine, and intensive care. To minimize complications such as arterial spasm, catheter kinking, or perforation, ultrasound-guided cannulation is strongly recommended [37,38]. In cardiac surgery, the RA is frequently utilized for CABG. Although the internal mammary artery is generally preferred, the RA demonstrates superior long-term patency and lower complication rates compared to saphenous or femoral vein grafts. Nevertheless, RA anatomical variations may still result in procedural challenges or failure [39,40].

Certain RA variants may confer clinical advantages. For instance, a recurrent RA may provide collateral blood flow in the event of surgical complications, making it particularly valuable in head and neck flap surgeries where anastomosis is required [30]. Overall, a thorough understanding of RA variants is critical across multiple medical specialties, as precise identification can improve procedural outcomes and reduce the risk of complications, underscoring the importance of detailed anatomical and imaging assessments during preoperative planning.

Although these variations may remain asymptomatic throughout life, their recognition is essential for interventions such as percutaneous coronary intervention in acute coronary syndrome or arterial catheterization in critically ill patients, where the radial approach is often preferred due to favorable clinical outcomes [41]. Anatomical variations in the RA pose a considerable challenge during catheterization, which can be mitigated through the use of ultrasound, the gold standard for ensuring procedural safety particularly when evaluating small or tortuous arteries in transverse sections [41]. Therefore, proficiency in imaging techniques, procedural skills, and a comprehensive understanding of RA anatomy are critical to achieving optimal outcomes and avoiding complications [42].

### 4.5. Embryological Association

The origin of RA anatomical variations including high origin, tortuosity, superficial course, or hypoplasia remains a subject of ongoing investigation. These variations are thought to arise from deviations in the development of the upper limb vasculature, often due to the persistence or incomplete regression of transient embryonic vascular connections [1]. Molecular signals, growth factors, and interactions between endothelial and smooth muscle cells may influence these processes. Notably, the RA is among the last arteries to complete its development in the forearm; for example, a high-origin RA may result from the persistence of an early anastomosis with the proximal brachial artery [43].

## 5. Limitations

Several potential limitations of this review should be acknowledged. As with any meta-analysis, the findings are inherently constrained by the publication and authorship biases presented within the included studies. First, research with differing or non-significant results that appeared in non-indexed or regional journals may have been unintentionally excluded, limiting the comprehensiveness of the data pool. Second, despite rigorous methodological efforts, limitations in search sensitivity and specificity could have resulted in the omission of relevant studies. Consequently, these factors may have increased the likelihood of missing data regarding unreported radial artery (RA) variants, particularly from regions outside Asia and North America, such as Oceania and Africa, where scientific reporting on this topic remains limited.

## 6. Conclusions

The radial artery (RA) exhibits a variety of anatomical variations, including differences in its origin, course, branching patterns, and tortuosity. While some of these variations may confer functional advantages, such as enhanced perfusion in specific circumstances, they are more commonly associated with challenges in both diagnostic and therapeutic procedures. These challenges range from routine clinical assessments, such as vital sign monitoring, to higher-risk interventions including arterial blood gas sampling, cannulation, fistula creation, catheterization, vascular grafting, and flap surgeries. Unrecognized RA variations can result in serious complications, including ischemia and, in extreme cases, limb loss.

Consequently, early identification of RA anatomical variations should be integrated as a standard protocol in relevant clinical settings. We recommend the use of routine preoperative imaging, such as ultrasound or computed tomography (CT) angiography, for procedures considered high-risk. Further research is necessary to expand current knowledge, optimize clinical decision-making, improve cost-effectiveness, and ensure that interventions are performed by healthcare professionals with adequate training and updated expertise.

## Figures and Tables

**Table 1:** Details of the search strategy.

Database	Search Strategy	Results	
		25 February 2020	25 July 2025
Medline	((((“radial artery” [MeSH Terms] OR (“radial” [All Fields] AND “artery” [All Fields]) OR “radial artery” [All Fields]) AND (“variation” [All Fields] OR “variations” [All Fields])) OR ((“aberrance” [All Fields] OR “aberrances” [All Fields] OR “aberrancies” [All Fields] OR “aberrancy” [All Fields] OR “aberrant” [All Fields] OR “aberrants” [All Fields] OR “aberrated” [All Fields] OR “aberrating” [All Fields] OR “aberration” [All Fields] OR “aberrational” [All Fields] OR “aberrations” [All Fields] OR “aberrator” [All Fields] OR “aberrators” [All Fields]) AND (“radial artery” [MeSH Terms] OR (“radial” [All Fields] AND “artery” [All Fields]) OR “radial artery” [All Fields]))) AND (“clin anat” [Journal] OR (“clinical” [All Fields] AND “anatomy” [All Fields]) OR “clinical anatomy” [All Fields])) NOT (“review” [Publication Type] OR “review literature as topic” [MeSH Terms] OR “review” [All Fields])	104	105
Wos	radial artery variations OR aberrant radial artery AND clinical anatomy NOT review	70	70
CINAHL	radial artery variations OR aberrant radial artery AND clinical anatomy NOT review	12	12
SCOPUS	radial artery variations OR aberrant radial artery AND clinical anatomy NOT review	100	101
Google Scholar	radial artery variations OR aberrant radial artery AND clinical anatomy NOT review	80	80
	Total	366	368

All searches were conducted on 25 July 2025.

**Table 2:** Characteristics and primary findings of the studies included in the present meta-analysis.

Author and Year	Geographic	*N* and Sample	*N* and Prevalence	RA Variant	Laterality of Variant	RA Type Variant or Classification	Sex and Age	Clinical Considerations
Nasr, 2012 [2]	Saudi Arabia	50 donors	8/50	Variations in branching pattern of RA	5 rights 3 left	The RA originated from the medial aspect of the upper third of BA The RA originated from the lateral of the upper part of BA	30 males 20 females Age: NA	Variation in branching pattern of RA has great importance in cardiac catheterization for angioplasty, pedicled flaps or arterial grafts, where any abnormal position or division should be identified before surgery. Thus, physicians should be aware of this variation before starting the procedure.
Numasawa et al., 2014 [12]	Japan	756 patients	68/756	Variation in the origin of Radioulnar loop and tortuous configuration	706 rights 50 lefts	Bifurcated BA proximal to the intercondylar line of the humerus is considered an abnormal origin of the RA	632 males 124 females Mean age: 67.6 to 11.5 years	Some patients with anatomic variation in RA had significantly larger ulnar arteries than RA. In some of these difficult transradial cases with anatomic variation, transulnar approach might be a safe alternative to transradial procedure if the reverse Allen test result is normal.
Polfer et al., 2018 [13]	United States	26 patients	10/26	Variations in RA origin	16 right 10 lefts	The RA originated in proximal BA The RA originated in mid-BA	12 males 14 females Mean age: 52 years	High-origin radial arteries may have variations in the density and adequacy of the most terminal part of the vessel and its branches, which increases the risk of ischemia. Alternatively, the proximal origin of the RA may affect distal perfusion through its impact on flow characteristics in the other vessels to the upper limb.
Park et al., 2022 [14]	South Korea	1 donor	1/1	Atypical topographic relationship RA	NA	Superficial RA runs over tendons (0.52%) Deep RA is thin and penetrates the tendons of the anatomical snuff-box	1 male 91 years old	Clinical classification of RA variants is vital as they are the main cause of technical failure when performing transradial catheterization. If well documented, problems with the transradial approach are minimized.
Dogan et al., 2005 [15]	Ankara, Turkey	16 patients	3/16	Severe RA calcification (1/16) and variation in forearm arterial anatomy	NA	Mild stenosis, occlusion and diffuse calcification of the distal RA	5 females 11 males Mean age: 60 years	A severely calcified RA is a risk factor because the long-term patency of this vessel is questionable. Few studies have examined the prevalence of pre-existing disease in this vessel, using Doppler ultrasound [Pola 1996, Ruengsakulrach 2001, Nicholosi 2002] or histologically [Kaufer 1997].
Singer et al., 2018 [16]	Graz, Austria	1 patient	1/1	Variant course of superficial palmar branch of RA	Right	Superficial palmar branch of RA superficial to thenar muscles	Female 17 years old	The vast majority of reports describing variant courses of the superficial palmar branch of RA are limited to post-mortem anatomical studies. Therefore, it can be assumed that irregular courses of the artery rarely cause clinical problems requiring intervention.
Rojas-Marte et al., 2015 [17]	NY, USA	1 patient	1/1	High takeoff RA	Right	Severe aortic stenosis	Female 63 years	Although vascular complications with TRA are less common than with TFA, the main problems include asymptomatic occlusion, spasm, and perforation of the RA, with the formation of hematomas and pseudoaneurysms. Other complications have also been described, such as compartment syndrome, perforation of arteries beyond the BA and catheter retention.
Venkatanarasimha, et al., 2007 [18]	United Kingdom	1 patient	1/1	Hypoplastic small caliber radial artery right	Right	Small caliber RA had a high proximal origin with relatively sharp angulation at its BA origin	Female 30 years old	A high origin RA is the most common variation, arising from the axillary artery in 4% and from the BA in 1.6%. Variations and absence of the RA in the forearm are less than 0.03%.
Barry, et al., 2023 [19]	Amiens, France	45 donors	1/45	Origin of RA	Right	High origin of RA “BRA”	28 males 17 females Average age 79.2 ± 9.2	This knowledge can ensure greater safety and reliability of the harvesting technique for use as a graft.
Haładaj, et al., 2018 [20]	Lodz, Poland	120 isolated upper limbs	11/120 BRA: 11 Axillary BRA: 2 BA BRA: 9 Anastomosis: 6/11, 6/120	BRA anatomical variations regarding: Origin, Presence, Anastomosis, Pattern of RRA	BRA: 6 Right, 5 Left Axillary BRA: 2 Right BA BRA: Dominant anastomosis: 1 Right Balanced: 1 Right and 1 Left (M), and 1 Right (F) Minimal: 1 Left (M), 1 Right (F) Abscent: 1 left and 1 right (M), 2 left and 1 right (F).	BRA in 9.2% (from axillary artery in 2 cases and from the BA in 9 cases). 54.6% anastomosis between BRA and BA in the cubital fossa, in 1 case, balanced in 3, minimal in 2 and absent in 5. division (trifurcation of the BA) in 9.2% (11/120), from the BRA in 5% (6/120) and from the cubital crossing in 4.2% (5/120).	ABR: 6M and 5F ABR axillary: 2M ABR BA: 4M and 5F Dominant anastomosis: 1M Balanced: 2M and 1F Minimal: 1M and 1F Absent: 2M, 3F 65M, 55F	The presence of BRA “contributed significantly to the development of tortuosity,” which may increase the risk of failure of transradial catheterization.
Li, et al., 2013 [21]	Guangxi, China	1400 patients	57/1400 origin 170/1400 with tortuosity	Tortuosity, Origin	NA	RA tortuosity (3.6%), ARB (1.7%), RA loop (0.6%) BA branches: Double RA (0.1%), double BA (0.1%)	1072 male 328 females age ≥18	Patients with tortuous RA tended to present with more classic chest pain. RA tortuosity and variation have become the leading cause of failure in coronary interventions through the RA, with the main factors for tortuosity being advanced age, female sex, short stature, etc.
Naman, et al., 2020 [22]	Xinjiang, China	1731 patients	86/1731	Origin, radioulnar loop and tortuosity.	NA	Variations origin of the RA (bifurcation of the BA proximal to the intercondylar line of the humerus)	1462 male 269 females Age: NA	Sex, occupation and internal diameter of the RA are significantly associated with the incidence of variation in the RA.
Yokoyama, et al., 2000 [23]	Tokyo, Japan	115 patients	11/115	Tortuosity, stenosis, hypoplasia of the RA and radioulnar loop.	NA	A tortuous RA was demonstrated on imaging in seven patients. Radial stenosis was observed (2/115)	83 males 32 females Mean age: 64.5 ± 9.7 years	Of the 115 patients undergoing TRI, 11 presented anatomical variations, of which 3 presented hypoplasia of the RA (1.7%) and a radioulnar loop (0.9%) making access of the guidewire and the introducer sheath impossible, which was finally planned using the femoral artery.
Vazquez, et al., 2013 [24]	United Kingdom	332 upper limbs from 166 donors separated into two groups	103/332 83 from group one 20 from group two	Accessory branch of the RRA	NA	The accessory branch of the RRA originated from the BA and ran behind the bicipital tendon, supplying the brachioradialis, brachialis, and biceps brachii muscles. The main RRA in group one originated from RA (75%), posterior radioulnar division (9%), anterior (5.4%), BA (7.2%), and ulnar-interosseous trunk (2.7%). The main RRA in group two originated from BRAl (65%).	79 male donors 87 female donors Age at death: Between 57 and 101 years.	Group one: normal pattern of the arteries of the upper limb, Group two: variations in the main arterial trunks of the upper limb
Alameddine, et al., 2004 [25]	Salem and Springfield, Massachusetts.	1	1	Accessory Branch or Duplication	Left	Accessory branch of the RA in the left arm, runs over the deep fascia of the forearm.	NA	In this case, we recommend that the incision be modified slightly and placed more medially along the medial border of the FCRM.
Belbl, et al., 2024 [26]	Czech Republic	1 volunteer	1	NA	Bilateral	PMA and superficial dorsal branch of RA Type 1, according to Miletin classification	1 Female 19 years old	Use of ultrasound before planning surgical procedures.
Zheng, et al., 2014 [27]	Nankai, Tianjin, China	1 patient	1	Bilateral congenital absence of the RA	Bilateral	Both arms without RA and the ulnar arteries were small, while the anterior interosseous arteries were the dominant artery	1 male 43 years old	Coronary angiography and percutaneous coronary intervention were performed through the BA, since transradial PCI failed.
Narayanan, et al., 2018 [28]	Puducherry, India	1 donor	1	BA bifurcation	Right	BA bifurcation into a common radial-interosseous trunk and a superficial ulnar artery.	Male 63 years old	The superficial position of the UA may allow surgeons to raise a free ulnar flap for head and neck reconstructive surgeries.
Nie, et al., 2009 [29]	Beijing, China	3000 patients	610/3000	Tortuosity, hypoplasia, radioulnar loop, origin, stenosis.	NA	RA: tortuous configurations (5.0%), hypoplasia (2.2%), radioulnar loop (1.1%) variations in origin (7. 7%), stenosis (1.4%);	M 65.9% (1977), F 34.1% (1023) 61 ± 8.1 years old	Anatomical variations in the RA are frequent and constitute an important limitation of the transradial approach.
Hamahata, et al., 2012 [30]	Japón	18 limbs from 18 donors	1/18	Variations in the origin of the RRA	NA	The anatomical variation in the RRA was divided into its origin into the RA (type A, 61.1%), RA bifurcation (type B, 33%), BA (type C, 0%) and UA (type D, 5%)	9 limbs from female donors 9 limbs from male donors Mean Age: NA	The use of radial forearm flaps plays an important role in head and neck operations. These involve performing anastomoses between the involved arteries where the RRA can be used instead of the RA to effectively transfer the flap.
Lee, et al., 1995 [31]	NA	1 patient	1/1	Aberrant RA	Left	An aberrant RA in the left arm of a patient with Klippel-Feil syndrome had progressive atrophy of the left thenar eminence	1 male patient 10 years old	Thoracic outlet syndrome should be considered for patients with KFS as compression of the subclavian artery may compromise blood supply and aggravate thenar atrophy.
Vollala et al., 2008 [32]	India	1 donor	1/1	Variation in the course of the RA	Right	A trifurcation of the BA in which the RA follows a variation in the course passing deep to the PTM	1 male donor 45 years old	Knowledge of arterial variations in the upper limb is necessary in research, orthopedic or surgical procedures and catheterization. The RA is used for cannulation, so its variation in course under the PTM can cause arterial compression and erroneous blood pressure values. The RA is used to replace the great saphenous vein as a CABG.
Schena et al., 2011 [33]	United States	25 volunteers (sub cohort)	25/25	Absence of RA after surgery	NA	Absence of RA implies increased blood flow to the UA and therefore its diameter, which can generate shear stress	21 males 4 females Mean age: 68.6 ± 8.6 years	In recent years, arterial grafts have been the main treatment for coronary artery disease, so the search for durable conduits to achieve better patency over time is of utmost importance.

Abbreviations: RA: Radial Artery; BA: Brachial Artery; BRA: Brachioradial Artery; CABG: Coronary Artery Bypass Grafting; F: Female; FCRM: Flexor Carpi Radialis Muscle; M: Male; NA: Not Available; PMA: Persistent Median Artery; PTM: Pronator Teres Muscle; TRI: Transradial Coronary Intervention; RRA: Radial Recurrent Artery; yo: Years-old; TRA: Trans-Radial Approach; TFA: Trans-Femoral Approach; KFS: Klippel-Feil.

## Data Availability

No new data were generated in this research. All data supporting the conclusions of this study are included in the published articles referenced throughout the manuscript.

## Supplementary Materials

The following supporting information can be downloaded at: https://www.mdpi.com/article/10.3390/diagnostics15232984/s1, Table S1: PRISMA 2020 Checklist [7].
